# Gastric Adenocarcinoma Associated with Acute Endocarditis of the Aortic Valve and Coronary Artery Disease in a 61-Year-Old Male with Multiple Comorbidities—Combined Surgical Management—Case Report

**DOI:** 10.3390/medicina55060242

**Published:** 2019-06-03

**Authors:** Horaţiu Moldovan, Daniela Popescu, Teodor Buliga, Anca Filip, Iulian Antoniac, Daniela Gheorghiţӑ, Adrian Molnar

**Affiliations:** 1 Department of Cardiovascular Surgery, Sanador Hospital, 010991 Bucharest, Romania; h_moldovan@hotmail.com; 2 Titu Maiorescu University, Faculty of Medicine, 004051 Bucharest, Romania; 3 Department of Cardiology, Sanador Hospital, 010991 Bucharest, Romania; dani31popescu@yahoo.com; 4 Department of General Surgery, Sanador Hospital, 010991 Bucharest, Romania; teodorbuliga@yahoo.com; 5 Department of Intensive Care, Sanador Hospital, 010991 Bucharest, Romania; anca.filip@sanador.ro; 6 University Politehnica of Bucharest, Faculty of Materials Science and Engineering, 060042 Bucharest, Romania; antoniac.iulian@gmail.com; 7 Iuliu Hatieganu University of Medicine and Pharmacy, 400129 Cluj–Napoca, Romania; adimolnar45@yahoo.com

**Keywords:** gastric tumor, gastrectomy, aortic valve endocarditis, acute pulmonary edema, double coronary lesions

## Abstract

The case of a 61-year-old male with a recent total gastrectomy for a hemorrhagic gastric tumor is presented, with the important co-morbidities of type II diabetes mellitus requiring insulin, chronic hepatitis C with liver dysfunction, stage II essential hypertension, chronic stage III renal disease peripheral type II aorto-iliac disease with stage II ischemia of both legs, and chronic anemia. About one month following the gastrectomy, the patient presented with fever and acute inflammatory syndrome. Severe aortic insufficiency, aortic valvular vegetations, and positive blood cultures with Staphylococcus saprophytic were found. The diagnosis of infectious endocarditis on the aortic valve was established (positive blood cultures with echocardiographic features of vegetations, fever), and antibiotic treatment with Levofloxacin and Vancomycin was initiated. The evolution was favorable with the remission of the inflammatory syndrome and quick cessation of fever. However, the hemodynamic aspect showed progressive heart failure with acute pulmonary edema. The transesophageal echocardiographic examination confirmed the existence of severe aortic insufficiency and valvular vegetations with a left ventricular ejection fraction of 38%. The coronary angiography revealed double vessel disease. The calculated Euroscore II was 33.4%. Aortic valve replacement with porcine xenograft and double coronary artery bypass graft surgery was performed. The patient had a favorable postoperative course remaining afebrile and out of heart failure, with the markers of inflammation largely within normal limits. The left ventricular ejection fraction increased to 50%. The successful outcome of this case, represented by a rare association of cancer, endocarditis, and coronary disease, reveals the importance of the multidisciplinary teams involved in this case: gastroenterology, general surgery, cardiology, infectious diseases, cardiac surgery, and intensive care. Therefore, in such cases with high risk, complex patients, a strong collaboration between all specialties is needed to overcome all of the limitations of the patient’s co-morbidities.

## 1. Introduction

This case report has a cardiological aspect, presenting a rare association between cancer and two cardiological aspects (coronary disease and endocarditis). Abdominal surgery complications were related in this case to cancer disease, that through its definition affects the immune system. With the immune system affected and preexistent co-morbidities, the patient developed a paraneoplastic immune depression. Therefore, the result was an infection with Staphylococcus saprophytic that represented the precursor to valvular endocarditis.

A frail patient subjected to an extensive surgical procedure can occasionally become subject to an unforeseen and unwelcome complication such as bacterial endocarditis. From mundane dental procedures or other types of maxillofacial surgery to any other surgical intervention of the gastrointestinal tract, bacterial or fungal endocarditis can follow [1,2]. Left sided endocarditis is more frequent than right heart endocarditis. Right heart valves are more frequently affected in patients on chronic hemodialysis, intravenous drug users, and patients with implantable electronic cardiac devices [3]. Left sided endocarditis is more common in patients with major comorbidities who undergo non-cardiac major surgery [4,5].

## 2. Case Presentation

**Figure 1:**
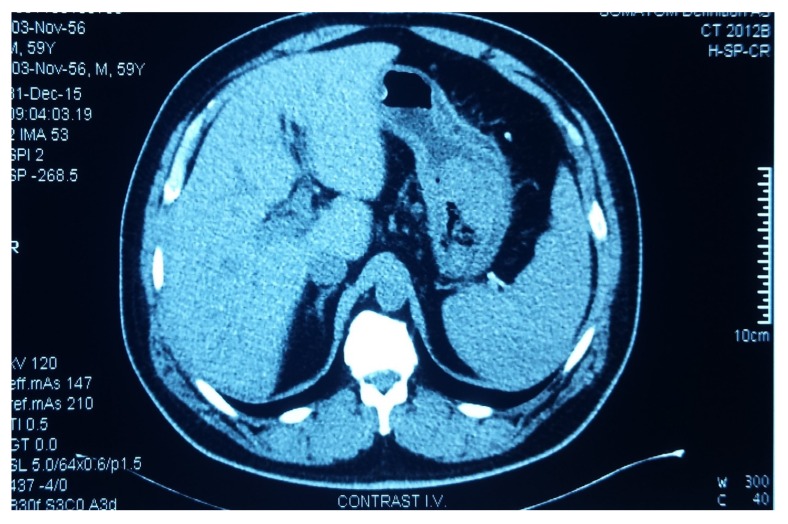
Computer Tomography (CT) scan showing the gastric tumor.

**Figure 2:**
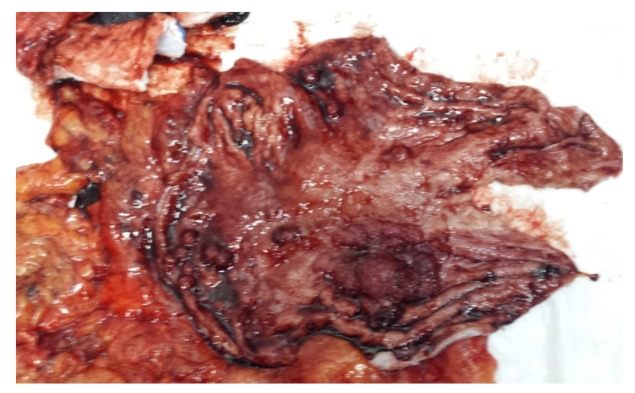
Surgical specimen with the gastric adenocarcinoma.

We present the case of a 61-year-old insulin-requiring diabetic patient with chronic C viral hepatitis, type II aorto-iliac peripheral artery disease with chronic stage II ischemia of both legs with anemia and hypovolemia who presented with a massive upper gastrointestinal tract (GIT) bleeding due to a hemorrhagic gastric adenocarcinoma. An urgent CT (computerized tomography) was performed, showing the gastric tumor (Figure 1). Based on the critical status of the initial presentation, the patient was taken to theater in emergency, where a total gastrectomy was performed (esophago-jejune-anastomosis, Omega shaped with Brown anastomosis at the tip of the jejunal loop) and the gastric adenocarcinoma surgical specimen was removed (Figure 2). After the gastrectomy procedure, the patient was closely monitored. Given the favorable evolution and lack of post-operative complications, the patient was discharged.

**Figure 3:**
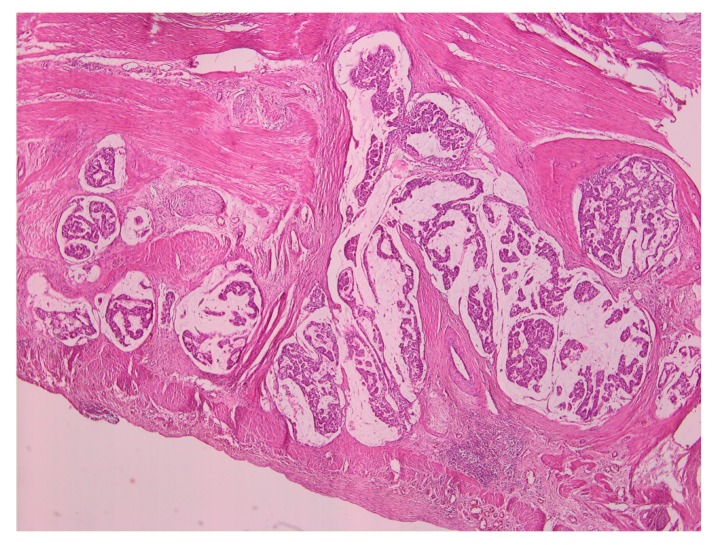
Histopathology examination of the gastric adenocarcinoma specimen.

The histopathological examination revealed the presence of regional lymphangiomas, some with moderately differentiated adenocarcinoma metastasis and the rest with only a reactive aspect. The examination further revealed tumor deposits in peritumoral adipose tissue, intramural intravascular tumoral emboli, and histologically preserved epiploon, without malignant tumoral elements. Throughout the gastric mucosa, at different levels, numerous formations of sessile and semi-pediculate grayish polyploids with a histopathological appearance of tubular adenomatous polyp and tubules with light and moderate dysplasia were found. Figure 3 presents a representative image of the histopathological examination.

**Figure 4:**
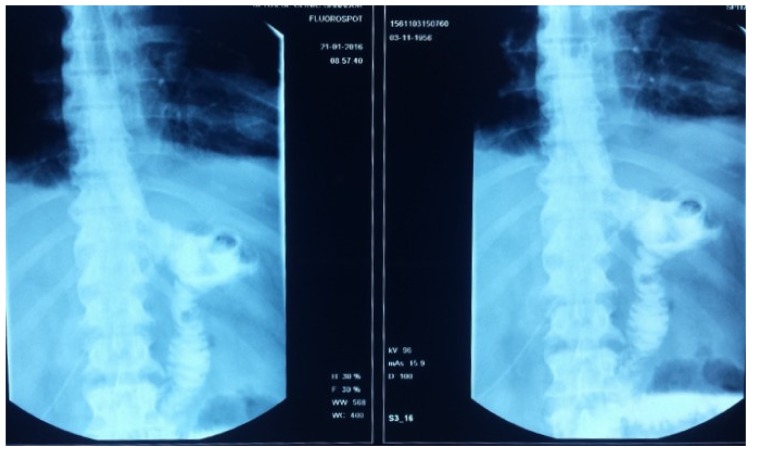
Radiological image after total gastrectomy and gastrojejunoanastomosis.

After the gastrectomy, the patient underwent X-ray radiography (Figure 4). Four weeks later, the patient developed a marked inflammatory syndrome (erythrocyte sedimentation rate (ESR) 100 mm, White Cell Count (WCC) 20,000, C-Reactive Protein test (CRP) 30, raised procalcitonin test, and pyrexia). The clinical examination revealed a diastolic aortic murmur with collapsing pulses. The transthoracic ECHO detected multiple aortic leaflet vegetations with severe regurgitation (grade IV). Blood cultures identified Staphylococcus saprophytic. As there was no existence of dental infections, malnutrition, or preexistent aortic lesions, due to the cancer and preexistent co-morbidities, the patient developed a paraneoplastic immune depression that resulted in the Staphylococcus saprophytic infection. 

Antibiotic treatment with IV Levofloxacin and Vancomycin was initiated, where there was a significant improvement of the inflammatory syndrome, however, the hemodynamic burden resulted in heart failure with acute pulmonary edema and recurrent episodes of myocardial ischemia at rest.

**Figure 5:**
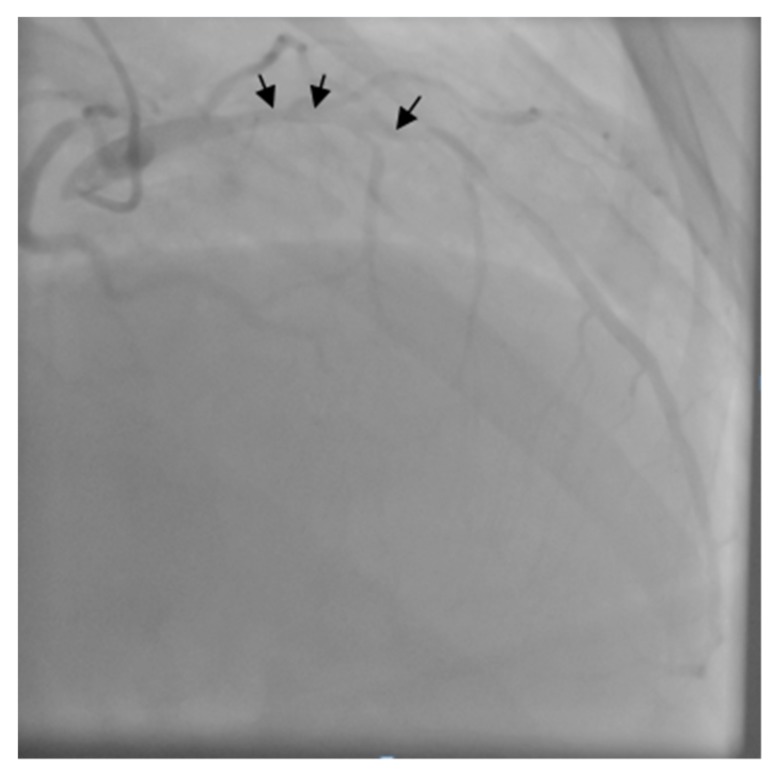
Left coronary angiogram - arrows show severe proximal left anterior descending artery (LAD) stenosis.

**Figure 6:**
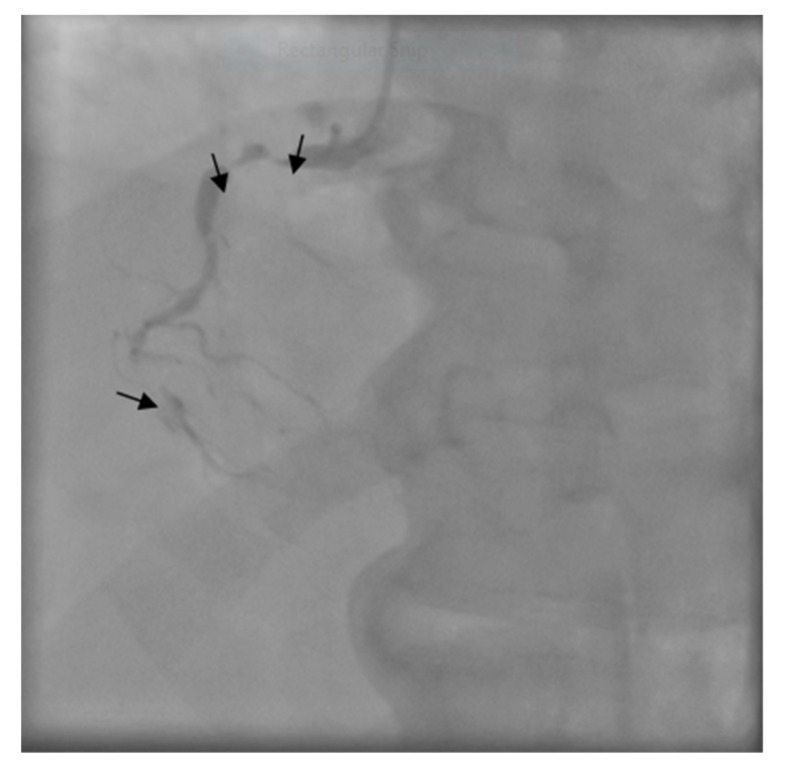
Right coronary angiogram - arrows show subocclusion in the second segment.

The patient was transferred to the Intensive Care Unit where he received inotropic support with Dobutamine, Levosimendan, and loop diuretics. The coronary angiogram showed a proximal critical stenosis of the left anterior descending artery (LAD) (Figure 5) and a tight lesion of the obtuse marginal (OM1) (Figure 6).

**Figure 7:**
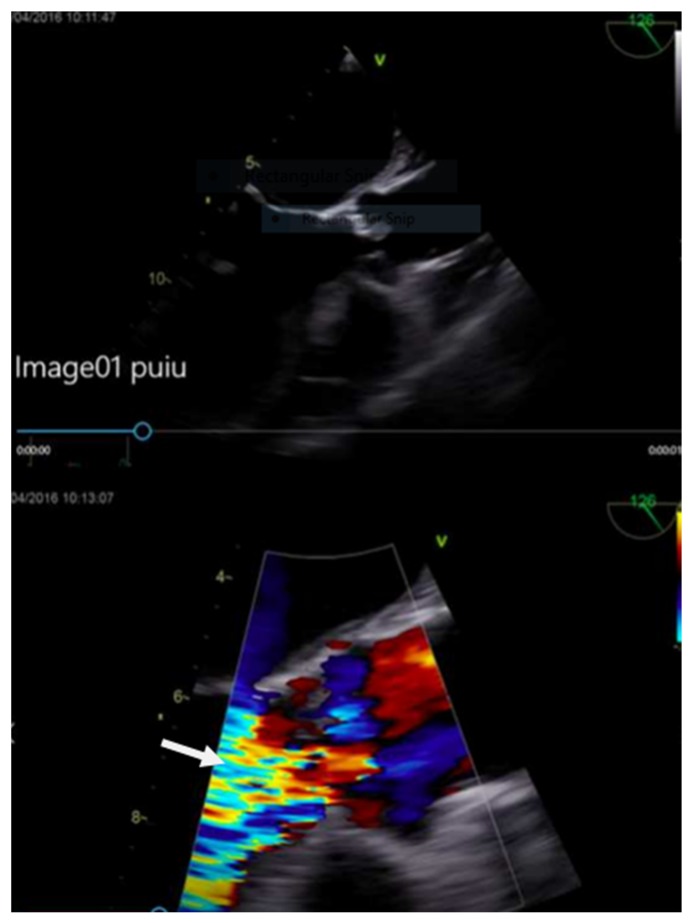
Echocardiographic image showing the aortic valve vegetation and aortic regurgitation.

The transesophageal echocardiography (TEE) showed a hypokinetic anterior wall of the left ventricle with significant systolic dysfunction ejection fraction (EF) 38%. There was a perforated noncoronary cusp with severe aortic regurgitation and a large 20 mm vegetation (Figure 7). The calculated Euroscore II was 33.4%.

**Figure 8:**
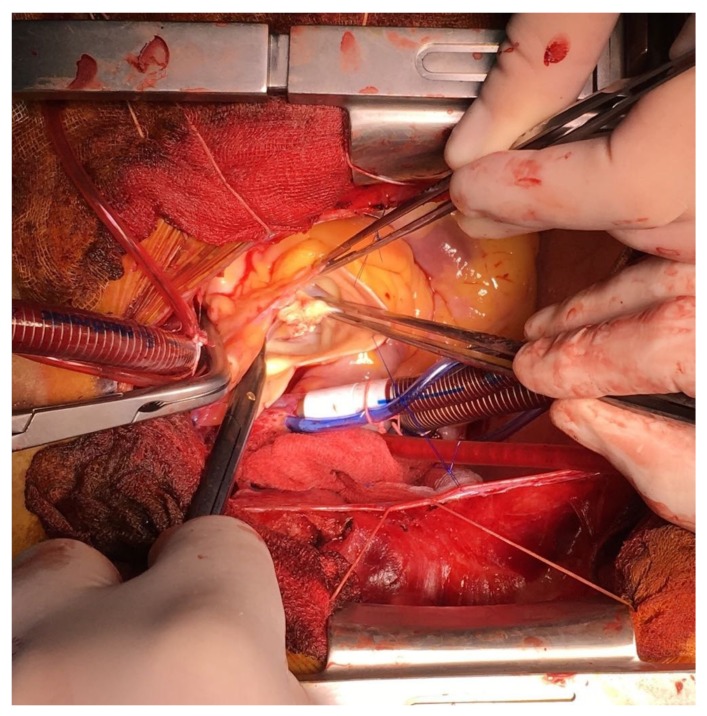
Surgical view of the aortic valve showing the aortic vegetation.

Aortic valve replacement (21 Medtronic Hancock II porcine xenograft) (Figure 8) and double vessel coronary artery bypass graft surgery was performed: left internal mammary artery (LIMA)—LAD and saphenous vein graft (SVG)—OM1. Intraoperatively, the perforation of the noncoronary cusp was confirmed and multiple vegetations were involved in all three aortic leaflets; fortunately there was no aortic root abscess. The study was approved under ethical number 3958 by Sanador Hospital (approved on 26 May 2016). Informed consent was obtained from the participant.

## 3. Discussion

The postoperative course was uneventful. The patient required mechanical ventilation for only 24 hours, and hemodialysis was necessary for a short period (three sessions only). Vasoconstrictor treatment with noradrenaline was continued for three days postoperatively and low dose dobutamine infusion was maintained for the entire period of stay in the Intensive Care Unit (ICU) stay.

Ten days following the cardiac surgery, the left ventricular ejection fraction increased to 45% and the inflammatory syndrome improved substantially. The patient, however, developed an extensive edematous syndrome two weeks later; hypoalbuminemia, heart failure, and inflammation could all have had a contributory factor. Diuretics, albumin infusion, and iron supplements were given and eventually the edema subsided.

The patient was discharged 18 days postoperatively in optimal conditions. The aortic prosthesis was functioning well, the left ventricular function greatly improved to a 50% ejection fraction without any sign of heart failure, there were normal levels of inflammatory markers, and no low grade pyrexia. Antibiotic treatment was continued for six weeks after surgery. The follow ups at one month, three months, and six months showed no further complications with a more than satisfactory clinical condition.

The appearance of a septic complication after cancer surgery can be represented by infectious endocarditis even if there have been no previous valvular lesions [6,7]. Usually, patients operated on for gastric cancer are closely monitored in a digestive surgery environment and the focus can resume on the abdominal sphere [8]. We therefore draw attention to a cardiac related complication after a major abdominal surgery [9,10].

## 4. Conclusions

Endocarditis with destruction of the aortic valve is a severe complication following major abdominal surgery for bleeding gastric adenocarcinoma. This patient had multiple co-morbidities: type II diabetes, liver dysfunction with hepatitis C, stage II essential hypertension, chronic stage III kidney disease, aortoiliac peripheral type II disease with chronic stage II ischemia of both legs, and chronic anemia.

The severity of the case was compounded by the ongoing myocardial ischemia due to critical coronary lesions (almost a left-main equivalent) and aortic regurgitation. The absence of cardiovascular symptoms before the abdominal surgery is puzzling in the context of anemia and hypovolemia. The successful outcome of this case with complex pathology is the direct result of a multidisciplinary approach where many hospital based specialties were involved.

This case report is a presentation of a rare case of cardiological complications associated with cancer. The complexity of this case is represented by the multiple diagnosis and co-morbidities that were successfully managed by the multidisciplinary team.
